# Stimulation of the Epithelial Na^+^ Channel in Renal Principal Cells by Gs-Coupled Designer Receptors Exclusively Activated by Designer Drugs

**DOI:** 10.3389/fphys.2021.725782

**Published:** 2021-08-25

**Authors:** Antonio G. Soares, Jorge Contreras, Crystal R. Archer, Elena Mironova, Rebecca Berdeaux, James D. Stockand, Tarek Mohamed Abd El-Aziz

**Affiliations:** ^1^Department of Cellular and Integrative Physiology, University of Texas Health Science Center at San Antonio, San Antonio, TX, United States; ^2^Department of Integrative Biology and Pharmacology, University of Texas Health Science Center at Houston, Houston, TX, United States; ^3^Zoology Department, Faculty of Science, Minia University, Minya, Egypt

**Keywords:** vasopressin, sodium excretion, sodium transport, hypertension, epithelial sodium channel

## Abstract

The activity of the Epithelial Na^+^ Channel (ENaC) in renal principal cells (PC) fine-tunes sodium excretion and consequently, affects blood pressure. The Gs-adenylyl cyclase-cAMP signal transduction pathway is believed to play a central role in the normal control of ENaC activity in PCs. The current study quantifies the importance of this signaling pathway to the regulation of ENaC activity *in vivo* using a knock-in mouse that has conditional expression of Gs-DREADD (designer receptors exclusively activated by designer drugs; GsD) in renal PCs. The GsD mouse also contains a cAMP response element-luciferase reporter transgene for non-invasive bioluminescence monitoring of cAMP signaling. Clozapine N-oxide (CNO) was used to selectively and temporally stimulate GsD. Treatment with CNO significantly increased luciferase bioluminescence in the kidneys of PC-specific GsD but not control mice. CNO also significantly increased the activity of ENaC in principal cells in PC-specific GsD mice compared to untreated knock-in mice and CNO treated littermate controls. The cell permeable cAMP analog, 8-(4-chlorophenylthio)adenosine 3′,5′-cyclic monophosphate, significantly increased the activity and expression in the plasma membrane of recombinant ENaC expressed in CHO and COS-7 cells, respectively. Treatment of PC-specific GsD mice with CNO rapidly and significantly decreased urinary Na^+^ excretion compared to untreated PC-specific GsD mice and treated littermate controls. This decrease in Na^+^ excretion in response to CNO in PC-specific GsD mice was similar in magnitude and timing as that induced by the selective vasopressin receptor 2 agonist, desmopressin, in wild type mice. These findings demonstrate for the first time that targeted activation of Gs signaling exclusively in PCs is sufficient to increase ENaC activity and decrease dependent urinary Na^+^ excretion in live animals.

## Summary

This study shows that targeted stimulation of Gs signaling in renal principal cells is sufficient to increase ENaC activity and decrease dependent Na^+^ excretion. Gs regulation of ENaC is critical to maximizing urine concentration and the normal control of blood pressure.

## Introduction

The epithelial Na^+^ channel (ENaC) is expressed in sodium (re)absorbing epithelial tissue to include that lining the aldosterone-sensitive distal nephron, which encompasses the late distal convoluted tubule, the connecting tubule and the collecting duct (Garty and Palmer, 1997; Rossier et al., 2002; Verouti et al., 2015; Hanukoglu and Hanukoglu, 2016). ENaC is restrictively expressed in the luminal plasma membranes of principal cells (PC) in the distal nephron. The activity of ENaC in the luminal plasma membrane of PCs is limiting for cell entry of Na^+^ from the urine and consequently, transepithelial Na^+^ reabsorption across the distal nephron (Kemendy et al., 1992; Canessa et al., 1993, 1994; Lingueglia et al., 1993). Ultimately, Na^+^ reabsorbed via ENaC fine-tunes serum Na^+^ levels, and contributes to the renal axial corticomedullary hypertonic gradient that concentrates urine (Reif et al., 1986; Perucca et al., 2008; Stockand, 2010). This makes the proper activity of ENaC critical to the normal control of blood pressure and for maximizing urinary water concentration (Bugaj et al., 2009). Because of this function, gain- and loss-of-function mutations in ENaC and its upstream regulatory pathways cause disordered blood pressure associated with abnormal renal salt excretion (Garty and Palmer, 1997; Rossier et al., 2002; Verouti et al., 2015; Hanukoglu and Hanukoglu, 2016). Consequently, this key renal ion channel is a druggable target for diuretics and antihypertensive agents to include triamterene and amiloride.

There is considerable evidence that G-protein coupled receptors (GPCRs) signaling through the Gs alpha subunit increase the activity of ENaC by stimulating adenylyl cyclase (AC) and dependent production of cAMP (Bugaj et al., 2009). The antidiuretic hormone, arginine vasopressin (AVP), is thought to increase ENaC activity in such a manner via the GPCR arginine vasopressin receptor 2 (AVPR2) to cause an anti-natriuresis along with its better understood antidiuretic ability (Perucca et al., 2008). Such activation of ENaC likely is key to AVP maximizing urine concentrating ability (Bugaj et al., 2009; Stockand, 2012; Mironova et al., 2012, 2015). While there is considerable evidence that Gs-coupled GPCR signaling is important to ENaC activity, the bulk of this evidence is correlative, derived from pharmacological intervention studies testing necessity, or from *in vitro* work. Moreover, the anti-natriuretic actions of AVP remain controversial (Stockand, 2010). Thus, it currently is obscure whether stimulating Gs signaling in isolation is sufficient to activate ENaC in living animals, and whether this alone is capable of inducing a quantifiable anti-natriuresis.

The current study tests the hypothesis that targeted stimulation of Gs in PCs is sufficient to increase ENaC activity in living animals and that such activation of ENaC is sufficient to drive decreases in urinary Na^+^ excretion. Moreover, these studies determined the mechanism by which Gs-cAMP signaling increases ENaC activity. The current findings demonstrate that exclusive activation of Gs signaling in renal PCs is sufficient to rapidly increase ENaC activity to decrease Na^+^ excretion. Gs signaling does this by promoting cAMP-mediated increases in ENaC expression in the plasma membrane, which increases channel activity.

## Materials and Methods

### Animals

All animal use and welfare adhered to the National Institutes of Health *Guide for the Care and Use of Laboratory Animals*. Protocols were reviewed and approved by the Institutional Animal Care and Use Committee of the University of Texas Health Science Center at San Antonio. Mice were housed and cared for in the Laboratory Animal Resources Facility at the University of Texas Health Science Center at San Antonio, which is fully accredited by the Association for Assessment and Accreditation of Laboratory Animal Care, and licensed by the United States Department of Agriculture. Results involving animal studies were in compliance with Animal Research: Reporting of *in vivo* Experiments guidelines (Kilkenny et al., 2010).

Healthy young adult (2–3 months, 20.61 ± 0.18 g body weight) male and female mice were used in approximately equal proportions for these studies. Experimental mice had PC-specific expression of GsD and control mice were either wild type, Aqp2-cre negative, or GsD negative littermates (see below). All mice, during acclimation and experimental periods, were maintained on a normal 12:12 h light-dark cycle at room temperature and had free access to water and chow. All experimental perturbations, including injections with CNO and D-Luciferin were performed within a constant time window (midmorning) in the laboratory where mice were housed. The primary end points for experiments involving mice in these studies were whole body bioluminescence in live animals, 24 h urinary excretion studies, and humane euthanasia followed by collection of kidneys for microdissection of renal nephrons compatible with electrophysiological assessment of ENaC activity in the split-open connecting tubule/cortical collecting duct.

### Creation of the Principal Cell-Specific Gs-DREADD Mouse

Target expression of Gs-DREADD (GsD) in renal PCs was achieved by crossing male *ROSA26-LSL-GsDREADD-CRE-luc* knock-in mice with female B6.Cg-Tg(Aqp2-cre)1Dek/J mice. With this knock-in allele, GsD is expressed in targeted cells following Cre-mediated recombination and deletion of the “lox-stop-lox” cassette (LSL), and cAMP-dependent signaling is reported by a cAMP-response element-sensitive luciferase (CRE-luc) independent of expression of Cre recombinase. The *ROSA26-LSL-GsDREADD-CRE-luc* knock-in mouse was created by Dr. R. Berdeaux and has been described previously (Akhmedov et al., 2017). The B6.Cg-Tg(Aqp2-cre)1Dek/J mouse was from Dr. D. Kohan (University of Utah Health Science Center, Salt Lake City, Utah, United States) and has been described previously (Nelson et al., 1998; Stricklett et al., 1999). The PC-specific GsD knockin line was continued by backcrossing GsD:Aqp2-cre female to GsD male mice. GsD:Aqp2-cre-positive mice were used for experiments. Wild type and Aqp2-cre-negative littermates lacking or harboring GsD were used as control. No noticeable difference in behavior, body weight, pathology, or any other gross attribute was observed between GsD:Aqp2-cre and littermate controls.

**FIGURE 1:**
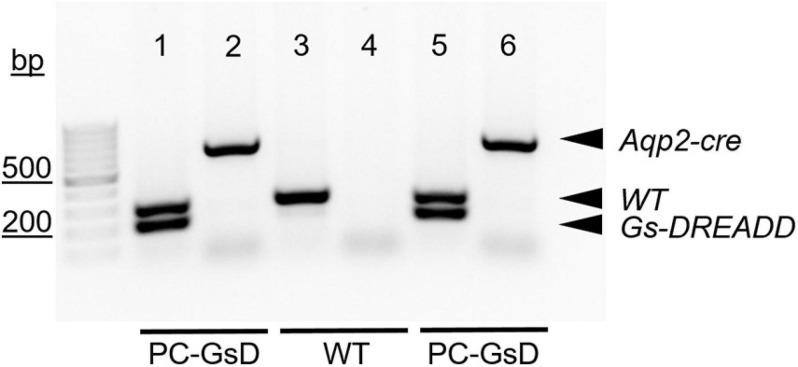
PC-specific GsD mice. Micrograph of a representative gel containing products from genotyping reactions for two PC-specific GsD mice (lanes 1, 2, 5, and 6) and one wild type littermate control mouse (lanes 3 and 4). Products for the Aqp2-cre and GsD transgenes, and the wild type allele are indicated with arrowheads. For presentation purposes, contrast and brightness were adjusted, and the image was inverted (black to white) to maximize clarity without changing content. bp, base pairs.

For genotyping reactions, like those shown in Figure 1, the GsD transgene was identified with the forward 5′-CTCGAAGTACTCGGCGTAGG-3′ and reverse 5′-CTCGAAGTACTCGGCGTAGG-3′ PCR primers, producing an expected 206-bp product. The wild type allele, which lacked insertion of the GsD transgene, was identified with the forward 5′-AAGGGAGCTGCAGTGGAGTA-3′ (in the upstream homology arm) and reverse 5′-CCGAAAATCTGTGGGAAGTC-3′ (in the downstream homology arm) PCR primers, producing an expected 297-bp product. The Aqp2-cre transgene was identified with the forward 5′-CTCTGCAGGAACTGGTGCTGG-3′ and reverse 5′-GCGAACATCTTCAGGTTCTGCGG-3′ PCR primers, producing an expected 673-bp product.

### cDNA Constructs and Cell Culture

African green monkey kidney fibroblast-like (COS-7) cells were purchased from ATCC (American Type Culture Collection, ATCC, Manassas, VA, United States). COS-7 cells were cultured in Dulbecco’s modified Eagle’s medium supplemented with 10% fetal bovine serum (FBS) and 1% penicillin-streptomycin (PS). CHO cells were cultured in Ham’s F-12K Nutrient Mixture (Kaighn’s Mod.) supplemented with 10% FBS and 1% PS. All cells were incubated at 37°C in a humidified incubator supplying 5% CO_2_. For TIRF-FRAP experiments (see below), COS-7 cells were plated on glass bottom MatTek dishes (35 mm petri dish-14 mm microwell, MatTek Co., MA, United States) and transfected with plasmids encoding α-, β-, and γ-mouse ENaC subunits genetically linked to NH_2_-terminal eYFP (Staruschenko et al., 2005) using Lipofectamine 3000 reagent (L3000015, Invitrogen, Carlsbad, CA, United States) according to the manufacturer’s protocol. In brief, 60% confluent cells in a 35 mm dish were incubated with 2 μg of total plasmid cDNA for 4–5 h. Media was then removed and replaced with fresh media with cells used within 24–48 h after transfection. For patch-clamp recording of macroscopic ENaC currents (see below), CHO cells were plated on coverglass chips treated with 0.01% Poly-L-lysine (Sigma-Aldrich, St. Louis, MO, United States). Plated cells were transfected with plasmids encoding α-, β-, and γ-mouse ENaC subunits genetically linked to NH_2_-terminal eCFP (Staruschenko et al., 2005) using Polyfect reagent (Qiagen, Valencia, CA, United States) according to the manufacturer’s protocol. In brief, 60% confluent cells in a 35 mm dish were incubated with 2 μg of total plasmid cDNA for 4–5 h, media was then removed and replaced with fresh media, and transfected cells used within 24–48 h. Transfected cells were maintained in culture in the presence of 10 μM amiloride replenished daily.

### Bioluminescence Reporter Imaging

Bioluminescence imaging was performed on mice before and 6 h after injection of CNO (10 mg/kg; s.c.) as previously described (Akhmedov et al., 2019). In brief, mice were anesthetized by isoflurane inhalation (2% v/v), injected (i.p.) with D-Luciferin (100 mg/kg), and transferred to the heated stage (37°C) of an Ivis Lumina XR Instrument (Capiler Life Science) for imaging (F-stop 1, binning 4). Image analysis was performed by selecting regions of interest to quantify luminescence signals using Caliper Living Image software. Pseudocolored images were overlaid onto photographs of the same animals and exported using matched visualization settings.

### Single-Channel Patch-Clamp Electrophysiology

Split-open tubules amenable to patch clamp analysis were prepared as previously described (Mironova et al., 2013, 2019). In brief, freshly isolated mouse kidneys were sectioned transversely. Segments of the CNT and CCD were manually microdissected with watchmaker forceps under a stereomicroscope and adhered to a glass chip coated with 0.01% poly-lysine. These chips then were transferred to an inverted microscope, where tubules were split open with sharpened pipettes. Gap-free single-channel patch-clamp electrophysiology in the cell-attached configuration (−V_p_ = −60 mV) was then performed on the luminal plasma membranes of PCs in these split-opened tubules following standard protocols (Bugaj et al., 2012; Stockand et al., 2012; Berman et al., 2018; Mironova et al., 2019). Split-open tubules were used within 1–2 h of isolation. The bath solution contained (in mM): 150 NaCl, 5 KCl, 1 CaCl_2_, 2 MgCl_2_, 5 glucose and 10 HEPES (pH 7.4); and the pipette solution (in mM): 140 LiCl, 2 MgCl_2_, and 10 HEPES (pH 7.4). Channel activity (NP_o_; open probability, P_o_, multiplied by channel number, N) was calculated as previously described (Stockand et al., 2012; Berman et al., 2018; Mironova et al., 2019). ENaC activity was recorded in tubules pre-treated with vehicle or CNO (2 μg/ml) for 30 min prior to patch clamp analysis.

### Total Internal Reflection Fluorescence Microscopy—Fluorescence Recovery After Photobleaching (TIRF-FRAP)

Total internal reflection fluorescence (TIRF) microscopy followed standard procedures (Staruschenko et al., 2005; Abd El-Aziz et al., 2021). In brief, fluorescence emissions from membrane eYFP-tagged ENaC were collected in COS-7 cells at room temperature using TIRF microscopy to selectively illuminate the plasma membrane. TIRF generates an evanescent field that declines exponentially with increasing distance from the interface between the cover glass and plasma membrane illuminating only a thin section (∼100 nm) of the cell in contact with the cover glass. All TIRF experiments were performed in the TIRF microscopy Core Facility housed within the Department of Cellular and Integrative Physiology at the University of Texas Health Science Center, San Antonio. Fluorescence emissions from fluorophore-tagged ENaC were collected using an inverted TE2000 microscope with through-the-lens (prismless) TIRF imaging (Nikon, Melville, NY, United States). This system is equipped with a vibration isolation system (Technical Manufacturing Co., Peabody, MA, United States) to minimize drift and noise. Samples were imaged through a plain Apo TIRF 60x oil immersion and high resolution (1.45 NA) objective. Fluorescence emissions from tagged subunits were collected with the Chroma Technology Co. (Bellows Falls, VT, United States) 514 nm laser filter set with band-pass emission (Z514BP) by exciting eYFP with an argon ion laser (80 mW) with an acoustic optic tunable filter (Prairie Technologies, Hutto, TX, United States) used to restrict excitation wavelength to 514 nm. In this system, a 514 nm dichroic mirror (Z514rdc) separates the 514/10 nm (Z514/10x) and 560/50 nm (HQ560/50m) excitation and emission filters. Fluorescence images were collected and processed with a 16-bit, cooled charge-coupled device camera (Cascade 512F; Roper Scientific, Sarasota, FL, United States) interfaced to a PC running Metamorph software (Molecular Devices, San Jose, CA, United States). This camera uses a front-illuminated EMCCD with on-chip multiplication gain. Images were collected with a 200 ms exposure time immediately before and after photobleaching and every subsequent minute for a total period of 10 min.

Fluorophore-tagged channels in the plasma membrane were photobleached with TIRF illumination using the argon ion laser (514 nm) at full power (100%) for 10 sec. Fluorescence emissions from membrane localized fluorophores were collected under TIRF illumination before and after photobleaching. Laser power, camera gain, and exposure times were constant throughout the course of these fluorescence recovery after photobleaching (FRAP) experiments except during photobleaching as noted above. All TIRF-FRAP experiments were performed 48 h after transfection.

### Whole-Cell Patch Clamp Electrophysiology

Whole-cell macroscopic current recordings of mouse ENaC expressed in CHO cells were performed under voltage clamp condition using standard methods (Staruschenko et al., 2006). In brief, current through ENaC was the macroscopic, amiloride-sensitive Na^+^ current with a bath solution of (in mM) 150 NaCl, 1 CaCl_2_, 2 MgCl_2_, and 10 HEPES (pH 7.4) and a pipette solution of (in mM) 120 CsCl, 5 NaCl, 2 MgCl_2_, 5 EGTA, 10 HEPES, 2 ATP, and 0.1 GTP (pH 7.4). Current recordings were acquired with an Axopath 200B (Molecular Devices, San Jose, CA, United States) patch-clamp amplifier interfaced via a Digidata 1550B (Molecular Devices, San Jose, CA, United States) to a PC running pClamp 11 software (Molecular Devices, San Jose, CA, United States). All currents were filtered at 1 kHz. Cells were clamped to a 40 mV holding potential with voltage ramps (500 ms) from 60 to −100 mV. Whole-cell capacitance, on average 8–10 pF, was compensated. Series resistance, on average 3–6 MΩ, was also compensated. Current conducted by ENaC was the amiloride-sensitive Na^+^ current.

### Metabolic Cages Experiments

Metabolic cage experiments quantifying excretion over a 24 h period followed previously published protocols with minor modifications (Mironova et al., 2015, 2019; Berman et al., 2018). In brief, age and weight matched PC-specific GsD and littermate control mice were housed in metabolic cages (1 mouse/cage; Techniplast, Buguggiate, Italy) and allowed to acclimate for 2 days. On the third day, 24 h urines were collected before CNO injection. Urine (24 h) was collected again on the fourth day following injection with CNO (0.1 mg/kg; s.c.). In a second set of experiments, wild type littermate mice were treated with the AVPR2 selective agonist, deamino-Cys,D-Arginine-vasopressin (dDAVP; desmopressin; 1 μg/100 μl water, i.p.), prior to collection of 24 h urine on the fourth day. Collection surfaces in contact with urine where coated with Sigmacote (Sigma-Aldrich, St. Louis, MO, United States), and urine was collect under light mineral oil to increase the precision of these measurements by reducing resistance to flow to the final collecting reservoir and to minimize loss due to evaporation. Urinary Na^+^ (U_Na_) and K^+^ (U_K_) concentration were quantified with a flame photometer (Jenway, Staffordshire, United Kingdom). Urinary osmolality was quantified with a vapor pressure osmometer (Wescor, Logan, UT, United States). Urinary [Na^+^] and [K^+^] were multiplied by 24 h urine volume (V) to obtain excretion.

### Statistical Analysis

Data were analyzed and plotted using GraphPad Prism 9 (GraphPad Software, Inc., San Diego, CA, United States). Values reported as mean ± standard error of the mean (SEM). Data were compared using a two-sample, two-tailed or paired *t*-test as appropriate, and a *P* ≤ 0.05 was considered significance.

## Results

### Targeted Stimulation of GsD Increases cAMP-Response Element Reporter Activity Selectively in the Kidneys of PC-Specific GsD Knockin Mice

**FIGURE 2:**
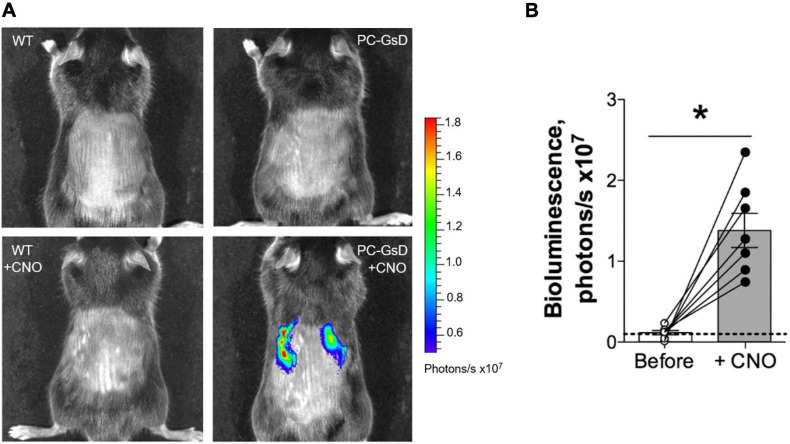
CNO stimulates cAMP-signaling exclusively in the kidneys of PC-specific GsD mice. **(A)** Micrographs showing representative *in vivo* bioluminescence (pseudo-colored for intensity) in control (left) and PC-specific GsD (right) mice before (top) and after (bottom) treatment with CNO (10 mg/kg, 6 h). **(B)** Summary graph of paired data for bioluminescence (photons/s × 10^7^) in PC-specific GsD mice before (white circles) and after (black circles) treatment with CNO. Bioluminescence in WT mice in the absence and presence of CNO was never greater than background (not shown). Dashed line indicates background level. Data from experiments (*n* = 7 mice) identical to those shown in **(A)**. *P < 0.05 vs. before.

We began these studies by testing whether targeted expression of GsD in PCs resulted in selective and temporal cAMP signaling in the kidney. *In vivo* bioluminescence responsive to signaling through the cAMP-response element (CRE) was evaluate in wild type littermate and PC-specific GsD mice before and 6 h after treatment with CNO. Figure 2A shows representative micrographs reporting CRE-sensitive bioluminescence in littermate controls (left) before (top) and after treatment with CNO (bottom), and in PC-specific GsD (right) mice before (top) and after (bottom) treatment with CNO. A summary graph of results from such experiments is shown in Figure 2B. As expected, no bioluminescence activity (above background) was observed in control mice in the absence (0.09 ± 0.05 photons/s ×10^7^) or presence (0.08 ± 0.04 photons/s ×10^7^) of CNO (not shown in summary figure; *n* = 7). In contrast, treatment with CNO significantly increased CRE-sensitive bioluminescence selectively in the kidneys of PC-specific GsD knockin mice from background levels of 0.12 ± 0.03 to 1.38 ± 0.29 photons/s ×10^7^ within 6 h of treatment.

### Targeted Activation of Gs-DREADD in Principal Cells Increases ENaC Activity

**FIGURE 3:**
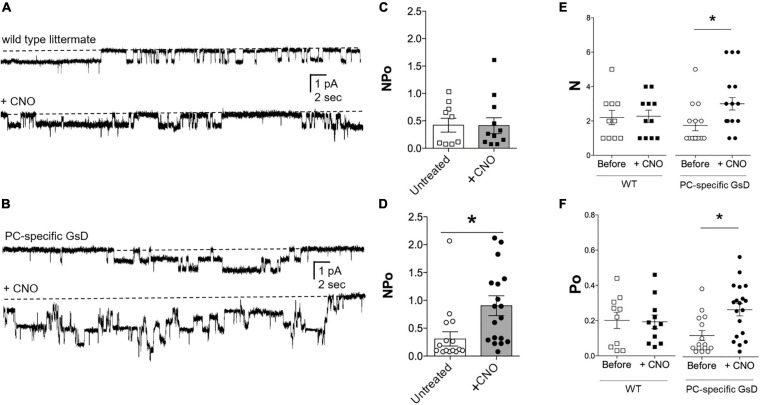
CNO increases ENaC activity in PC-specific GsD mice. Representative gap-free current traces of ENaC in cell-attached patch clamp recordings of the apical membranes of PCs in isolated tubules from wild type littermates **(A)** and PC-specific GsD **(B)** mice in the absence (top) and presence (bottom) of pre-treating tubules with CNO (2 μg/ml, 30 min). Inward Na^+^ current is downwards with dashed lines indicating the closed state. Summary graphs of ENaC activity (NP_o_) in PCs in tubules isolated from wild type controls **(C)** without (white boxes, *n* = 9 patches from 3 distinct mice) and with CNO (black boxes; *n* = 11 patches from 3 distinct mice), and from PC-specific GsD mice **(D)** without (white circles, *n* = 15 patches from 3 distinct mice) and with CNO (black circles, *n* = 18 patches from 3 distinct mice). *P < 0.05 vs. no CNO treatment. Summary graphs of ENaC N **(E)** and P_o_ **(F)** in PCs in tubules isolated from wild type controls (white boxes, *n* = 10 from 3 distinct mice) and after CNO (black boxes, *n* = 10 from 3 distinct mice); and from PC-specific GsD mice before (white circles, *n* = 13 from 3 distinct mice) and after CNO (black circles, *n* = 10 from 3 distinct mice). Summary data from experiments identical to those shown in **(A,B)**. *P < 0.05 vs. Before.

We tested next whether selective stimulation of Gs signaling in PCs was sufficient to increase ENaC activity. ENaC activity (NP_o_) in the apical plasma membranes of PCs in tubules isolated from littermate control and PC-specific GsD mice was quantified in cell-attached patches using patch clamp electrophysiology. Figure 3 shows representative current traces for ENaC in tubules isolated from control (Figure 3A) and PC-specific GsD (Figure 3B) mice before (top) and after (bottom) treatment with CNO. Figures 3C–F summarize all such results. Figure 3C shows that ENaC activity (NP_o_) in tubules from control mice was unaffected by treatment with CNO (0.42 ± 0.12 vs. 0.41 ± 0.14). In contrast, CNO significantly increased ENaC activity in PCs in tubules isolated from PC-specific GsD mice from 0.31 ± 0.13 to 0.90 ± 0.18 (Figure 3D). The activity of ENaC in tubules from PC-specific GsD mice in the absence of CNO was not different than that in littermate controls. Increases in both ENaC number (N; 1.73 ± 0.30 vs. 3.00 ± 0.36; Figure 3E) and open probability (P_o_; 0.12 ± 0.03 vs. 0.27 ± 0.03; Figure 3F) drove activity increases in PC-specific GsD mice in response to CNO treatment.

### cAMP Signaling Increases ENaC Expression in the Plasma Membrane, in Part, to Increase Channel Activity

**FIGURE 4:**
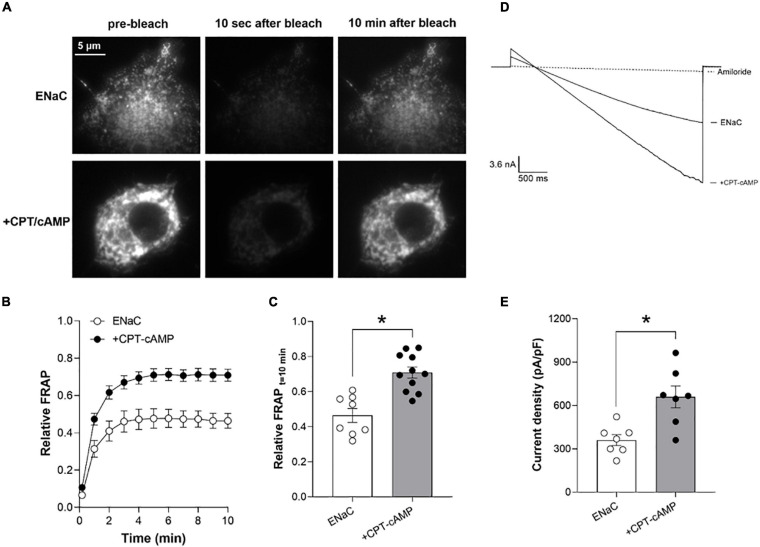
cAMP signaling increases ENaC expression in the plasma membrane to increase channel activity. **(A)** Representative fluorescence micrographs of the plasma membranes of COS-7 cells expressing recombinant eYFP-ENaC in the absence (top) and presence of CPT-cAMP (bottom) prior to photobleaching (left) and 10 s (middle) and 10 min (right) after photobleaching. Images were collected with TIRF microscopy. **(B)** Time courses of relative FRAP at the plasma membrane for cells expressing eYFP-ENaC in the absence (white circles, *n* = 8) and presence of CPT-cAMP (200 mM; black circles; *n* = 11). Summary data from experiments identical to those shown in **(A)**. **(C)** Summary graph of relative FRAP 10 min after photobleaching in cells expressing eYFP-ENaC in the absence (white circles, white bar) and presence of CPT-cAMP (black circles, gray bar). Summary data from experiments identical to those shown in **(A)**. *P < 0.05 vs. no CPT-cAMP. **(D)** Overlays of typical macroscopic current traces from representative CHO cells expressing recombinant mouse ENaC in the absence and presence of CPT-cAMP (200 mM) and after adding 10 mM amiloride (dotted lines). Currents elicited by voltage ramps stepped from a holding potential of 40–60 mV and ramped to –100 mV. **(E)** Summary graph of ENaC activity (amiloride-sensitive current density at –100 mV) quantified in whole-cell voltage clamped CHO cells transfected with mouse ENaC in the absence (white circles, white bar) and presence (black circles, gray bar) of CPT-cAMP treatment. Summary data from experiments (*n* = 7) identical to those shown in **(D)**. *P < 0.05 vs. no CPT-cAMP.

Figure 4A shows representative fluorescence micrographs of the plasma membranes of COS-7 cells expressing eYFP-tagged ENaC in the absence (top) and presence (bottom) of treatment with CPT-cAMP (8-(4-chlorophenylthio)adenosine 3′,5′-cyclic monophosphate) before (left), 10 s after (middle) and 10 min after (right) photobleaching. Figures 4B,C show the time-course and magnitude, respectively, of the relative recovery (normalized to pre-bleach levels) of ENaC in the plasma membrane following photobleaching in the absence (white circles) and presence (black circles) of CPT-cAMP. Treatment with cAMP significantly increased relative FRAP of ENaC in the plasma membrane at 10 min from 0.46 ± 0.05 to 0.71 ± 0.05.

Similarly, treatment with CPT-cAMP increased the activity (amiloride-sensitive current density at −100 mV) of recombinant ENaC expressed in CHO cells from 360.6 ± 33.1 in untreated cells to 660 ± 84.2 pA/pF in treated cells. Figure 4D shows overlays of representative macroscopic ENaC currents in CHO cells in the absence and presence of CPT-cAMP, and amiloride (dashed lines). Results from these experiments are summarized in Figure 4E.

### Targeted Activation of Gs-DREADD in Principal Cells Decreases Na^+^ Excretion

**FIGURE 5:**
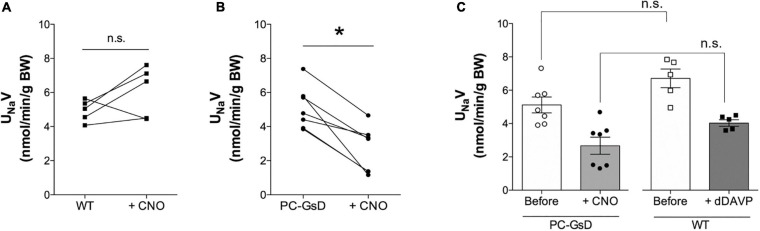
CNO increases urinary Na^+^ excretion in PC-specific GsD mice. Summary graphs from paired experiments showing 24 h urinary Na^+^ excretion (U_Na_V) before and after treatment with CNO (0.1 mg/kg) in wild type littermate controls (**A**, *n* = 5) and PC-specific GsD mice (**B**, *n* = 7). *P < 0.05 vs. before treatment. **(C)** Summary graph of 24 h urinary Na^+^ excretion before (white circles, white bar) and after (black circles, gray bar) CNO treatment for PC-specific GsD mice, and control mice before (white boxes, white bar) and after (black boxes, dark gray bar) dDAVP treatment (1.0 mg/100 ml; *n* = 5).

To understand the physiological consequences of Gs-stimulation of ENaC in PCs in the living animal, we quantified CNO-sensitive urinary Na^+^ excretion (U_Na_V) in control littermates and PC-specific GsD mice. Figures 5A,B show summary results from paired experiments quantifying U_Na_V before and after treatment with CNO in control (Figure 5A) and PC-specific GsD (Figure 5B) mice. Sodium excretion was not significantly affected by CNO in control mice (4.94 ± 0.28 vs. 6.06 ± 0.70 nmol/min/g BW). In contrast, CNO significantly decreased U_Na_V in PCs-specific GsD mice from 5.12 ± 0.48 to 2.67 ± 0.51 nmol/min/g BW. Sodium excretion in PCs-specific GsD mice before treatment with CNO was not different from that in untreated control mice. Figure 5C shows summary results for U_Na_V in PCs-specific GsD mice before and after treatment with CNO, and control littermates before and after treatment with the selective AVPR2 agonist, desmopressin. As expected, desmopressin significantly decreased sodium excretion from 6.72 ± 0.95 to 4.06 ± 0.23 nmol/min/g BW. Sodium excretion in PC-specific GsD with CNO, though, was not different from that in wild type mice with dDAVP at these doses and times. Similarly, U_Na_V in PC-GsD mice prior to treatment with CNO did not differ from that in untreated control mice. These summary results demonstrate that the effects on sodium excretion of CNO in PCs-specific GsD mice are similar to that of desmopressin in control mice with respect to timing and magnitude.

Table 1 shows summary results for U_K_V and osmolality in WT and PC-specific GsD mice before and after treatment with CNO. Potassium excretion was not significantly affected by CNO in control mice (11.20 ± 1.3 vs. 10.37 ± 1.4 nmol/min/g BW). In contrast, CNO significantly increased U_K_V in PCs-specific GsD mice from 14.27 ± 1.5 to 31.49 ± 4.7 nmol/min/g BW. Osmolality was not significantly affected by CNO in control mice (1,799 ± 105 vs. 1,836 ± 90.78 mmol/kg). CNO treatment significantly increased urine osmolality in PCs-specific GsD mice from 1,997 ± 119 vs. 2,438 ± 109 mmol/kg. These effects on potassium excretion and osmolality in the PCs-specific GsD mice are consistent with the actions of vasopressin and with effects on U_Na_V, and consistent with targeted activation of ENaC in PCs of the distal nephron.

**TABLE 1:** Urinary potassium excretion (U_*K*_V) and osmolality in PC-specific GsD mice.

	**U_*K*_V (nmol/min/g BW)**		**Osmolality (mmol/kg)**	
	**Before**	**After CNO**	**Before**	**After CNO**
WT	11.20 ± 1.3	10.37 ± 1.4	1,799 ± 105	1,836 ± 90.78
PC-specific GsD	14.27 ± 1.5	31.49 ± 4.7*^,#^	1,997 ± 119	2,438 ± 109*^,#^

*P < 0.05 vs. WT; ^#^P < 0.05 vs. PC-specific before CNO.

## Discussion

These studies demonstrate that targeted stimulation of Gs signaling in renal PCs is sufficient to increase ENaC activity and decrease dependent urinary Na^+^ excretion in live animals. This is important to understanding the role played by Na^+^ reabsorption through ENaC in maximizing urine concentrating ability and control of blood pressure.

Arginine vasopressin has well understood antidiuretic action. In comparison, the consequences and underpinnings of potential anti-natriuretic effects of this hormone are more controversial (Stockand, 2010). AVP activates the Gs-coupled AVPR2 in renal PCs which stimulates AC to increase cellular cAMP levels. Nine AC isoforms have been identified but only three are expressed in renal PCs: AC3, AC4 and AC6 (Strait et al., 2010). Whereas AC3 and AC6, but not AC4, are necessary for some portion of AVP-dependent increases in cellular cAMP levels in inner medullary collecting duct cells, AC6, but not AC3, is necessary for AVP effects on ENaC in PCs in the intact mouse tubule (Strait et al., 2010; Roos et al., 2013; Kittikulsuth et al., 2014). Consequently, deletion of AC6 but not AC3 specifically in PCs results in a urine concentrating defect in mice that mimics global knockout of AC6 and nephrogenic diabetes insipidus (Rieg et al., 2010; Roos et al., 2012; Kittikulsuth et al., 2014). Gs-AC signaling ultimately increases ENaC activity by increasing cellular cAMP levels, and the Na^+^ reabsorbed from the urine through cAMP-stimulated ENaC adds to the axial corticomedullary hyperosmotic gradient that allows maximal urinary concentration in the presence of AVP-stimulated AQP2 channels (Bugaj et al., 2009; Stockand, 2010; Mironova et al., 2012, 2015).

The products of genotyping reactions shown in Figure 1 demonstrate that our PC-specific GsD mice are of the correct genotype. The *in vivo* bioluminescence results in Figure 2 demonstrate the expected functional consequences of appropriate recombination upon crossing the GsD knock-in line with an Aqp2-cre driver line: restrictive and temporal expression of CNO-sensitive cAMP-signaling in the kidneys. This is consistent with cursory immunofluorescence studies showing GsD expression in ENaC expressing PCs in the distal nephron (see Supplementary Figure 1). Together this evidence demonstrates that the PC-specific GsD mouse used in these studies is as expected and expresses functional GsD in renal PCs.

Results in Figure 3 demonstrate that targeted activation of Gs selectively in PCs in tubules from this PC-specific GsD mouse rapidly and robustly increases the activity of ENaC by increasing both the number of channels expressed in the apical plasma membrane and also the open probability these channels have. Such observations are in agreement with earlier work showing that AVP increases ENaC activity in PCs in native tubules in an AVPR2-dependent manner (Bugaj et al., 2009; Mironova et al., 2012). Such AVP-stimulation of ENaC contributes to maximizing urinary concentration and dilution of plasma (Bugaj et al., 2009). These findings in the current study provide novel independent evidence that direct stimulation of Gs in PCs is sufficient to increase ENaC activity. This complements the existing knowledge that AVPR2-Gs-AC6-cAMP signaling is necessary for AVP stimulation of ENaC in PCs. Thus, evidence in support of necessity and sufficiency now exists.

The cellular mechanism by which Gs signaling increases ENaC activity is thought to be post-translational, culminating in changes in trafficking (and open probability) that result in increases in the levels of active channel in the plasma membrane (Snyder, 2000; Snyder et al., 2004; Fakitsas et al., 2007). Current findings as presented in Figures 3, 4 are consistent with this. We find that a cell permeable analog of cAMP that is resistant to hydrolysis, CPT-cAMP, markedly increases the levels of ENaC in the plasma membrane. While increasing cAMP levels with CPT-cAMP in the immortalized cells used in the current study to investigate mechanisms affecting activity of overexpressed ENaC is not a direct measurement of how stimulating Gs *in vivo* would affect ENaC, it is a reasonable surrogate for this that likely mimics with some fidelity how ENaC responds to stimulation of GsD in the living animal. Indeed, results from single channel patch clamp experiments on CNO-treated tubules from PC-specific GsD mice are in agreement that increases in channel number contributes to this mechanism.

Results in Figure 5 demonstrate that targeted stimulation of GsD in PCs temporally decreases renal Na^+^ excretion in live animals in a manner that is similar to the effects of desmopressin, an AVPR2 specific agonist. Such findings again demonstrate sufficiency, elaborating the physiological consequences of selective activation of Gs signaling and dependent stimulation of ENaC in PCs in the living animal. This novel finding is direct evidence that Gs signaling in PCs is anti-natriuretic. Elevated ENaC activity is the mechanistic underpinnings of this anti-natriuresis. Such findings add strong evidence to the argument that AVP has an anti-natriuretic effect in addition to its antidiuretic actions.

Together the anti-natriuretic and antidiuretic actions of AVP (via AVPR2-Gs-AC6-cAMP-ENaC/AQP2 signaling) contribute to maximal urine concentration and regulation of blood pressure (Stockand, 2010). The current findings provide the first direct evidence that stimulating Gs with subsequent activation of ENaC is sufficient to contribute to this action of AVP in the living animal.

## Data Availability Statement

The original contributions presented in the study are included in the article/Supplementary Material, further inquiries can be directed to the corresponding author/s.

## Ethics Statement

Protocols were reviewed and approved by the Institutional Animal Care and Use Committee of the University of Texas Health Science Center at San Antonio.

## Author Contributions

CA, EM, AS, JS, and TA conceived and designed the experiments. CA, RB, EM, JC, AS, and TA created the reagents and performed the experiments to collect the data. AS, TA, and JS created the manuscript figures and wrote the first draft of the manuscript, which was subsequently edited and approved by all authors.

## Conflict of Interest

The authors declare that the research was conducted in the absence of any commercial or financial relationships that could be construed as a potential conflict of interest.

## Publisher’s Note

All claims expressed in this article are solely those of the authors and do not necessarily represent those of their affiliated organizations, or those of the publisher, the editors and the reviewers. Any product that may be evaluated in this article, or claim that may be made by its manufacturer, is not guaranteed or endorsed by the publisher.
