# Supplementary figures and images for: Stimulation of the Epithelial Na+ Channel in Renal Principal Cells by Gs-Coupled Designer Receptors Exclusively Activated by Designer Drugs
Source: Front Physiol. 2021 Aug 25;12:725782. doi: 10.3389/fphys.2021.725782 (PMC8425396; doi:10.3389/fphys.2021.725782)

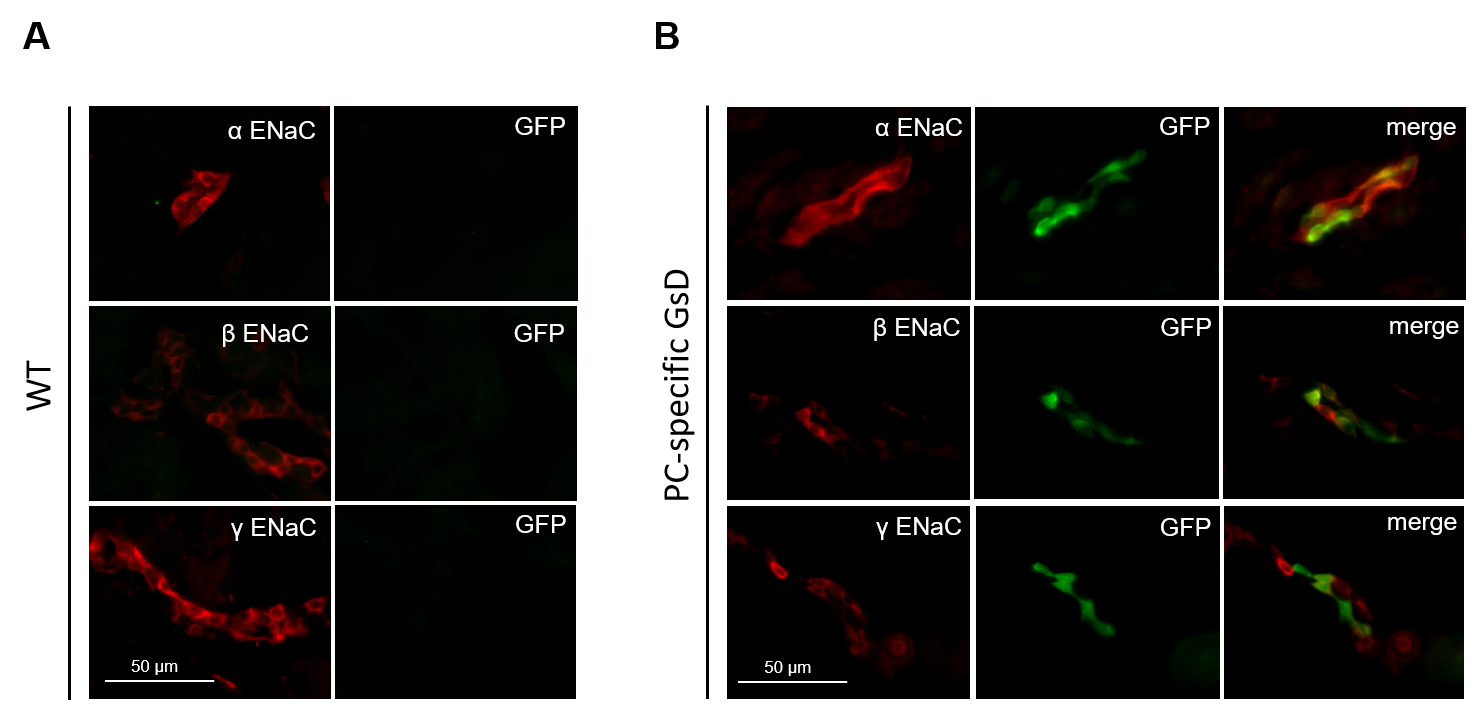

Supplement: Supplementary file 1 [file Image_1.TIF]
