# Supplementary material for: Stimulation of the Epithelial Na+ Channel in Renal Principal Cells by Gs-Coupled Designer Receptors Exclusively Activated by Designer Drugs
Source: Front Physiol. 2021 Aug 25;12:725782. doi: 10.3389/fphys.2021.725782 (PMC8425396; doi:10.3389/fphys.2021.725782)
Supplement: Supplementary file 2 [file Table_1.DOCX]

**Supplement Material**

**Material and Methods**

**Immunofluorescence**

Kidneys isolated from control and PC-specific GsD mice were perfused with ice-cold PBS and fixed in 4% paraformaldehyde at 4 °C overnight. Tissue was then transferred to a 30% sucrose solution at 4 °C overnight. After fixation, tissue was placed in cryomolds, frozen and sliced into 8 μm sections. Kidney slices were probed with rabbit anti-alpha (20341, BiCell Scientific, MO, USA), -beta (20342, BiCell Scientific, MO, USA) and -gamma (20343, BiCell Scientific, MO, USA) ENaC antibodies diluted 1:100. Goat anti-GFP antibody (600-101-215, Rockland, Limerick, PA, USA) was used at a 1:200 dilution. Slices were incubated with primary antibodies (described above) at 4 °C overnight. Then, slices were washed and incubated for 1 h with appropriate secondary antibody (Alexa Fluor 488-anti-goat IgG and Alexa Fluor Cy5-anti-rabbit IgG, Thermo Scientific, Waltham, MA, USA). Immunofluorescence images were collected on an Axiovert 200M microscope (Carl Zeiss, Thornwood, NY) interfaced to a PC running Slide Book 5 software (Intelligent Imaging Innovation, Inc, Denver, CO, USA).
