# Supplementary material for: Stimulation of the Epithelial Na+ Channel in Renal Principal Cells by Gs-Coupled Designer Receptors Exclusively Activated by Designer Drugs
Source: Front Physiol. 2021 Aug 25;12:725782. doi: 10.3389/fphys.2021.725782 (PMC8425396; doi:10.3389/fphys.2021.725782)
Supplement: Supplementary file 3 [file Table_2.DOCX]

**Supplement Figure 1. Gs-DREADD is expressed in principal cells.** Representative fluorescence micrographs of cortical renal sections from control (**A**) and PC-specific GsD (**B**) mice stained with anti-GFP (green, right) and anti-ENaC subunit (red, left), α (top), β (middle), and γ (bottom), antibodies. Merged image shown at right for (**B**). For representation purposes, sharpness, contrast, and brightness were adjusted to maximize clarity without changing content.
